# Workplace activity classification from shoe-based movement sensors

**DOI:** 10.1186/s42490-020-00042-4

**Published:** 2020-06-24

**Authors:** Jonatan Fridolfsson, Daniel Arvidsson, Frithjof Doerks, Theresa J. Kreidler, Stefan Grau

**Affiliations:** 1 Center for Health and Performance, Department of Food and Nutrition, and Sport Science, University of Gothenburg, Box 300, 405 30 Gothenburg, Sweden; 2 Hochschule Koblenz, University of Applied Sciences RheinAhr Campus, Remagen, Germany; 3 Institute for Applied Movement Science, Chemnitz University of Technology, Chemnitz, Germany

**Keywords:** Accelerometry, Workload, Physical activity, Occupational health

## Abstract

**Background:**

High occupational physical activity is associated with lower health. Shoe-based movement sensors can provide an objective measurement of occupational physical activity in a lab setting but the performance of such methods in a free-living environment have not been investigated. The aim of the current study was to investigate the feasibility and accuracy of shoe sensor-based activity classification in an industrial work setting.

**Results:**

An initial calibration part was performed with 35 subjects who performed different workplace activities in a structured lab setting while the movement was measured by a shoe-sensor. Three different machine-learning models (random forest (RF), support vector machine and k-nearest neighbour) were trained to classify activities using the collected lab data. In a second validation part, 29 industry workers were followed at work while an observer noted their activities and the movement was captured with a shoe-based movement sensor. The performance of the trained classification models were validated using the free-living workplace data. The RF classifier consistently outperformed the other models with a substantial difference in in the free-living validation. The accuracy of the initial RF classifier was 83% in the lab setting and 43% in the free-living validation. After combining activities that was difficult to discriminate the accuracy increased to 96 and 71% in the lab and free-living setting respectively. In the free-living part, 99% of the collected samples either consisted of stationary activities or walking.

**Conclusions:**

Walking and stationary activities can be classified with high accuracy from a shoe-based movement sensor in a free-living occupational setting. The distribution of activities at the workplace should be considered when validating activity classification models in a free-living setting.

## Background

Industry work is associated with a high physical workload. Although leisure-time physical activity is associated with health, the opposite is true with physically active works [1]. High occupational physical activity (OPA) is associated with more long term sickness absence as well as all-cause mortality [1, 2]. OPA is also a factor contributing to fatigue, which in a work setting could lead to serious injury or death [3]. Consequently, assessment of OPA would provide indication of detrimental volume, allowing appropriate adjustments of work tasks before fatigue occurs.

Objective monitoring of physical activity is widespread in research; occupational, epidemiological and clinical [4, 5]. However, the most common sensor positions are the hip or thigh, which are unpractical for monitoring OPA over time among industry workers. Since industry work often require safety shoes, because of e.g. toe protection and slip resistance, a sensor built into these shoes would be a practical and relatively easily implementable solution in an occupational setting. Previous work has shown that an accelerometer placed on a shoe has similar accuracy in predicting activities as other placements [6], but the performance of shoe based sensors have not been evaluated in a free-living setting previously [7].

Sensor based physical activity classification is usually based on data recorded by one or more accelerometers [4]. The raw acceleration signal is processed to display specific features. Features comprise for instance mean acceleration, max frequency, correlation between axes that are calculated continuously over a moving window. These features are then used for activity classification, either using simple empirically derived decision trees [8], or using statistical methods, called machine-learning, that is more common today [9]. Machine-learning algorithms can be developed to be extremely accurate in a structured setting reaching 98–100% accuracy [9, 10]. However, when applying the algorithms to other structured datasets (lab-data) the accuracy drops significantly [11], and with free living datasets, the accuracy is reduced even further [12].

The aim of the current study was to investigate the feasibility and accuracy of shoe sensor-based activity classification in an industrial work setting. Therefore, in a first step, shoe acceleration data was captured in a structured setting (standardized lab-activities) to develop an activity classification machine-learning algorithm that will be able to reliably distinguish between different activities. In a second step, the algorithm was validated in a free-living setting (at the workplace).

The following research questions were examined in this study:

1. Is it possible to reliably classify work specific activities with acceleration signals captured from a shoe-based sensor?
2. Does this activity classification work in a free-living (workplace) setting?

## Results

The study consisted of two parts, a calibration part in a controlled setting and a validation part in a free-living workplace setting. Thirty-five subjects participated in the calibration part. Information about the test subjects can be seen in Table 1.

**Table 1:** Subjects details

	N (% female)	Age (SD)***	BMI (SD)***
Lab calibration	35 (49%)	25.4 (6.0)	23.1 (2.3)
Free-living validation	29 (10%)	38.7 (11.5)	26.8 (3.5)

BMI: Body Mass Index, SD: Standard Deviation, *** indicates significant group differences *p* < 0.001.

**Fig. 1:**
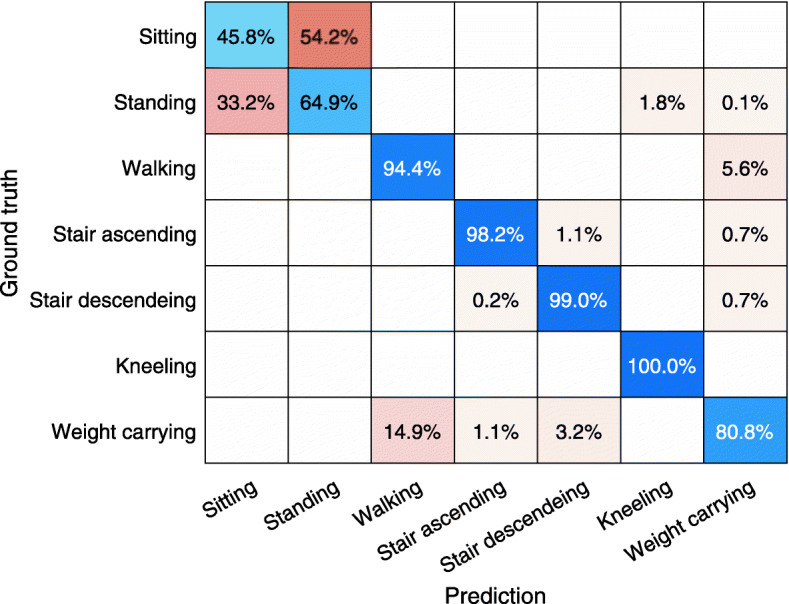
Performance of initial random forest classifier in the lab calibration setting. The numbers in the chart are row normalized, showing the distribution of the classification of the samples from each activity according to the test protocol. Y-axis shows the activity performed according to the test protocol. X-axis shows the predicted activity from the trained classification model

Subjects wore an accelerometer attached to their right shoe while performing seven standardized activities. The accelerometry data was processed to signal features used to train three different machine learning classification models to classify the standardized activities. The classification models used were random forest (RF), support vector machine (SVM) and k-nearest neighbour (KNN). The lab accuracy of the initial classification models are presented in Table 2 (Lab calibration 7 activities). The RF model accuracy was slightly higher than the other two models, but all within one percentage point. The activity specific performance of the RF model is presented as a confusion chart in Fig. 1. The distribution of accuracy between the different activities were similar between the three models. The RF accuracy of the different activities (blue marked) range from 45.8% (sitting) to 100% (kneeling). The model had difficulties differentiating standing and sitting (64.9 and 45.8% respectively). The classification accuracy of weight carrying was also less accurate (80.8%). 54.2% of sitting was misclassified as standing, 33.2% of standing was misclassified as sitting and 14.9% of weight carrying was misclassified as walking.

**Table 2:** Accuracy of models

	Random forest	Support vector machine	K-nearest neighbour
Lab calibration 7 activities	83.3%	82.3%	82.5%
Lab calibration 5 activities	96.3%	95.0%	95.7%
Free-living validation 7 activities	43.0%	35.7%	29.6%
Free-living validation 5 activities	71.2%	67.1%	63.4%

**Fig. 2:**
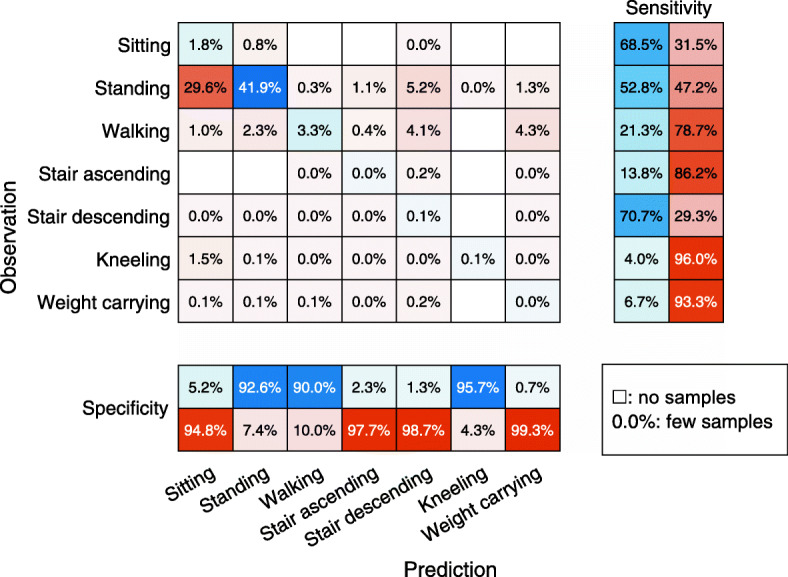
Performance of initial random forest classifier in the free-living workplace setting. The numbers in the chart are relative to the total number of samples. This implies that both the accuracy and distribution of observed and predicted samples can be identified from the chart. Y-axis shows the observed activity. X-axis shows the predicted activity from the classification model trained on lab data. The two columns to the right indicate the activity specific sensitivity (the proportion of observed samples classified correctly) and the two rows at the bottom indicates the activity specific specificity (the proportion of classified samples in agreement with the observation)

Twenty-nine subjects took part in the validation part of the study. The age and body mass index (BMI) of the subjects in the validation part was significantly different from the subjects in the in-lab calibration part (Table 1). Fifteen subjects were working at a logistics centre warehouse and 14 subjects were working in industrial production. An average of 48 min of free-living data was captured for each participant using an accelerometer attached to their right shoe. The performance of the three classification models in the free-living setting is presented in Table 2. Similar to the lab results, the RF model had the best accuracy, but in the free-living validation the differences between methods are much larger with 7.3 and 13.4 percentage points for SVM and KNN respectively. The activity specific performance of the RF model is presented as a confusion chart in Fig. 2. Numbers inside the chart are normalized to the total number of samples and shows the distribution of activities in addition to the accuracy. The activity specific sensitivity (the proportion of observed samples classified correctly) ranged from 4.0% (kneeling) to 60.0% (stair descending) and the activity specific specificity (the proportion of classified samples in agreement with the observation) ranged from 0.7% (weight carrying) to 87.7% (walking). Similar to the lab-performance, sitting and standing was difficult to differentiate. In contrast to the lab-results, most of the observed stair ascending were classified as stair descending. The specificity of walking was high (87.7%) but the sensitivity was low (22.4%). This was because many of the observed walking samples were classified as either stair descending or weight carrying but samples that were classified as walking mainly consisted of observed walking.

**Fig. 3:**
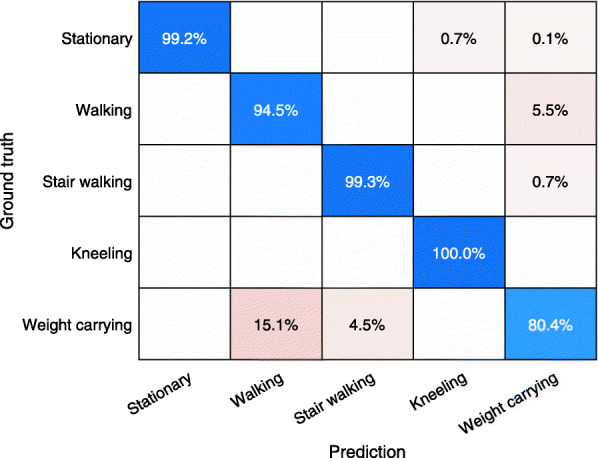
Performance of second random forest classifier in the lab calibration setting with sitting and standing combined to stationary as well as stair ascending and descending combined to stair walking. The numbers in the chart are row normalized, showing the distribution of the classification of the samples from each activity according to the test protocol. Y-axis shows the activity performed according to the test protocol. X-axis shows the predicted activity from the trained classification model

**Fig. 4:**
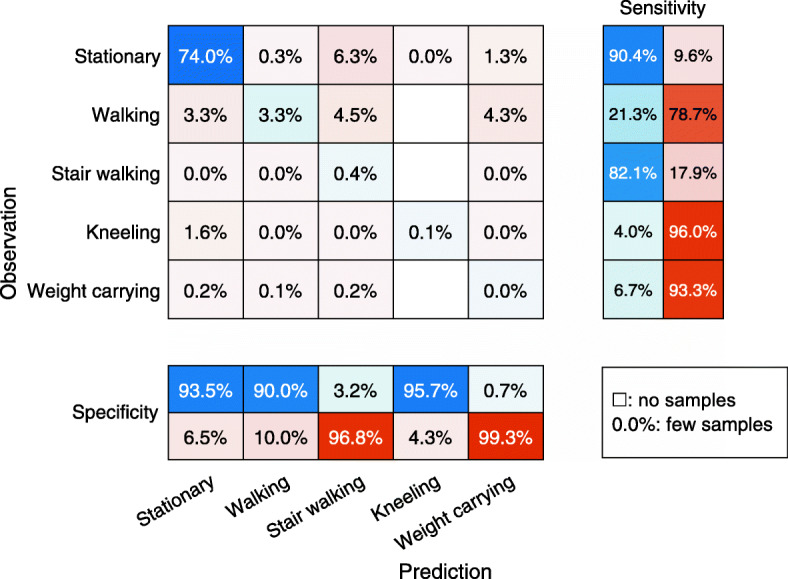
Performance of second random forest classifier in the free-living workplace setting with sitting and standing combined to stationary as well as stair ascending and descending combined to stair walking. The numbers in the chart are relative to the total number of samples. This implies that both the accuracy and distribution of observed and predicted samples can be identified from the chart. Y-axis shows the observed activity. X-axis shows the predicted activity from the classification model trained on lab data. The two columns to the right indicate the activity specific sensitivity (the proportion of observed samples classified correctly) and the two rows at the bottom indicate the activity specific specificity (the proportion of classified samples in agreement with the observation)

Three additional calibration models based on five activities were developed by using the same calibration data but combining sitting and standing into stationary and combining stair ascending and descending into stair walking. Similar to the initial seven activities models, the RF model outperformed the SVM and KNN models in lab and free-living validation (Table 2). The activity specific performance of the second RF model is presented in Fig. 3 (lab) and Fig. 4 (free-living). The classification accuracy of the in lab weight carrying was still less accurate (80.4%). In free-living, the predictions of the second model was highly accurate (88–91%) with regard to stationary and walking activities. These activities made up 99% of the collected samples. However, the performance on the rest of the activities were very low. To investigate the effect of activity type in the validation data, a sub analysis was performed on the industrial production and logistics warehouse data separately. With the industrial production data the accuracy of the seven and five activities models was 40.4 and 66.9% respectively. The accuracy was higher with the logistics warehouse data at 47.2 and 77.8% with the seven and five activities models respectively.

## Discussion

The RF classification model consistently outperformed the KNN and SVM models. The difference between models were negligible in the calibration setting but increased drastically in free living validation. The lab-setting RF model classification accuracy of activities at the workplace was consistently high except for standing and sitting (Fig. 1). In the free-living setting on the other hand, the classification accuracy was initially low across all activities (Fig. 2). After combining standing and sitting to stationary activity as well as combining stair ascending and descending to stair walking, the level of accuracy for both activities increased in the lab and free-living environment (Fig. 3-4). The good overall performance of the second RF model in the free living (71%) can be explained as 99% of the samples captured consisted of either walking or stationary activity.

Combining sitting and standing and not being able to distinguish between the two might be considered a major shortcoming of the classification model. On the other hand, although there is a small increase in energy consumption from standing compared to sitting [13, 14], standing is still considered a sedentary activity [15] and there are no cardiovascular health benefits with standing compared to sitting [16]. During both sitting and standing the feet are usually parallel to the ground and no movement occurs. Since the inclination and movement of the sensor is used for classification, this explains the difficulty discriminating the two stationary activities. Stair ascending and descending were also combined to a single activity in the second model. However, these activities are associated with significantly different energy expenditure as opposed to sitting and standing [14]. The estimated workload from stair walking might therefore be underestimated. Although, in most cases, stair descending and ascending could be assumed to be equally distributed.

The initial classification models’ performance were poor for all free-living activities (Fig. 2). The reason only standing/sitting and stair ascending/descending was combined was that these activities was clearly mixed up with each other but not with any other activity. With the other activities, the misclassification was more spread out. The differentiation between walking and stationary activities could probably just as well have been performed using an acceleration intensity metric alone [4]. However, activity type might be a more applicable output for the workplace than the abstract intensity measures.

Other weaknesses of the study are the significant sex, age and BMI differences between subjects in the two study parts. The participants in the validation group consisted of more men, were older and had higher BMI than the participants in the calibration group, which might have affected the classification performance. It should also be considered that the validation was performed indoors only whereas parts of the calibration was performed outdoors. Although the outdoor walking in the calibration part was also done at slow pace, most of the indoor walking in the validation part might have been done at even slower pace. Calibration of stair walking and weight carrying was performed indoors and at slower speeds than the normal walking speed, which could explain the misclassification of free-living walking into these activities (Fig. 4). The workers in the logistics warehouse were covering larger areas while walking, whereas the workers in productions were mainly walking a few steps between machines. Covering larger areas could make the difference between walking and stationary more prominent and explain the higher accuracy in with the logistics warehouse data.

Although direct observation is considered the criterion method for activity classification in a free-living setting [4], this method is not perfect. It has been shown that direct observation has an accuracy of 87% where the activity classification of senior researchers was considered the reference [17]. In a free-living setting, the activities might not be equivalent to the standardized lab-activities. Most of the validation data consisted of standing work that was stationary most of the time with walking a few steps in between (Fig. 2), which could be difficult to define with the current classification scheme.

Many studies on accelerometer based machine-learning classification models have been published previously, most of them using similar techniques as the current study [9, 18]. We have only found one other study that investigated the performance of a lab calibrated machine-learning method in a free living setting and there the accuracy was 49–55% [12]. The accuracy of the current study is substantially higher at 71% (Fig. 4) although it is very low with some activities. However, the activities classified are different in the two studies. Lab calibration of activity classification may be prone to overfitting, even when validating the model using leave one subject out cross validation [18]. Nevertheless, RF classification models are in general relatively robust to over fitting, but on the other hand may perform poorly on data that deviate much from the training data [19]. The main limitation in generalization of lab developed activity classification models is thought to be the diverse activity types, different characteristics within each activity type and individual variation [18]. The limited number of samples with other activities than stationary and walking at the two workplaces in the validation part limits further analysis of the accuracy of the classification of these activities. The classification model might perform better in a setting where other activities are more common. The difference in accuracy between workers in the logistics warehouse and industrial production also supports this assumption. A more diverse free-living dataset with a more even activity distribution could also be used to improve the classification algorithm further by analysing the temporal structure of activities [20].

Certain work-related physical activity patterns are suggested to have a negative health effect. For example, prolonged physical activity elevates 24-h heart rate and static postures and lifting is suggested to elevate 24-h blood pressure [21]. Such patterns might be detected by the activity classification system suggested in this paper. Continuous monitoring of workload among industrial workers could be used in many ways for preventive measures and improving health. The monitoring gives basic data on the distribution of physical workload across different tasks at the workplace. This can be utilized when partitioning tasks between workers, both with regard to sharing heavy work between more employees and to lower the physical demand on specific individuals. Continuous monitoring also provides the possibility to follow workload over time, which might enable identification of employees getting fatigued at an early stage. This could potentially result in an overall less long-term sickness absence and better health status among workers [1]. However, constant monitoring of activities during work do raise concerns regarding privacy of the workers [5].

## Conclusion

The results of the study shows that walking and stationary activities can be classified with high accuracy from a shoe based accelerometer. In order to accurately classify other activities (kneeling, stair ascending and descending and weight carrying), workplaces with a higher number of those activities should be considered. However, the present study also addresses issues with activity distribution when classifying activities in a free-living setting. It highlights difficulties with free-living validation of activity classification with regard to generalisation of lab-calibrated classification, observation of activities and activity dispersion in free-living. Despite this, the results suggest that activity classification using shoe-based sensors could give accurate and comprehensive feedback on walking and stationary activities in an occupational setting.

## Methods

Subjects for the calibration part were recruited through e-mail announcements to students and staff at the Department of Food and Nutrition and Sport Science and personal communication. Written informed consent was retrieved from the subjects and the study was approved by the regional ethics committee in Gothenburg (no. 765–18).

For the data collection, subjects wore safety shoes (Ergo-Active Grant, Elten GmbH, Uedem, Germany) in their respective size (EU 36–48) and width (narrow, medium, wide). Accelerometers (AX3, Axivity Ltd., Newcastle upon Tyne, UK) were then firmly attached to the heel-cap of each shoe orthogonal to the outsole using non-elastic adhesive tape. The accelerometers were set to record triaxial acceleration at a sampling frequency of 100 Hz and a range of +/− 16 *g,* where 1 *g* is equivalent to the gravitational acceleration. These specifications were sufficient to capture all acceleration related to human movement [22].

The test protocol of the calibration part consisted of eight activities that were performed by the subjects continuously for 1–4 min.

- Sitting on a chair, while solving Sudoku on a table
- Standing, while solving Sudoku on a high table
- Walking slow, self-paced, outdoors
- Walking brisk, self-paced, outdoors
- Stair ascending
- Stair descending
- Weight carrying while walking, 15 kg
- Kneeling

**Fig. 5:**
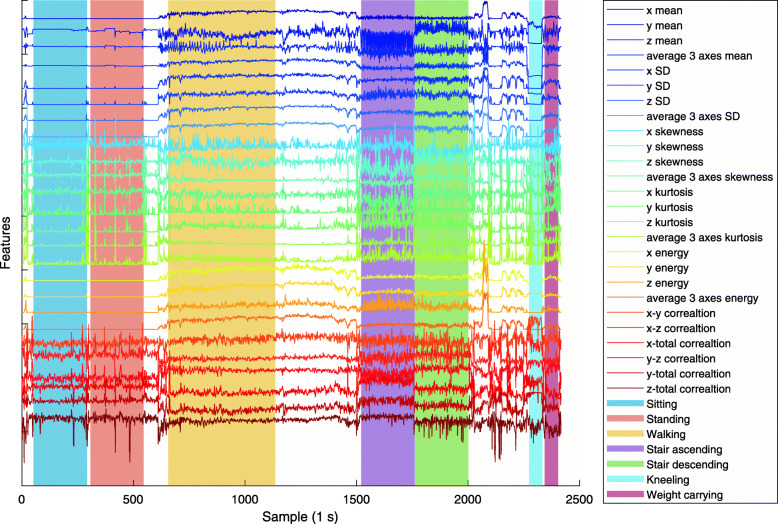
Example data from one subject showing features and activity type from lab calibration. Features are standardized to their respective mean and standard deviation

All analyses were performed in MATLAB R2018b (MathWorks, Natick, MA, USA). Acceleration was captured between the 6th and 55th second of the last minute of each activity. Using a 2 s window with 50% overlap [23], 26 signal features [6] were calculated for the extracted acceleration for each shoe. The features from each window were labelled according to the test protocol. Features and activity type from one subject are presented as an example in Fig. 5. Samples with outliers were removed from the data from each activity using a criterion of more than three scaled median absolute deviations (MATLAB rmoutliers-function). The labelled data was used as training data to generate three different machine-learning classification algorithms, random forest (RF), support vector machine (SVM) and k-nearest neighbour (KNN). These are among the most commonly used techniques for classifying activity based on accelerometer data [9, 18]. The models were implemented as an ensemble of bagged decision trees (RF), third degree support vector machine (SVM) and the ten nearest neighbours weighted by the inverse distance squared (KNN) using the MATLAB Classification Learner. Validation of the classification algorithms was performed by leave-one-out validation for each subject. This validation technique implies that there will never be data from the same subject in both the training- and validation data set simultaneously which leads to a more realistic accuracy measure [24].

Recruitment of subjects for the free-living validation part took place at an industrial workplace through information to the workers. Written informed consent was retrieved from the workers who agreed to participate. Subjects were fitted with the same kind of shoes as in the calibration part with the same accelerometers attached to the heel-cap. Then the subjects were told to work as usual while followed by an observer. The observer noted the start, end and type of each activity continuously for about 60 min. Since separating standing and walking could be difficult during standing work, walking was considered continuous movement more than three meters. This way a few sidesteps during standing work would still be considered a standing activity.

The collected accelerometer data was processed as in the calibration part of the study. The different features were input to the developed classification algorithms to get a predicted activity for each window. The observed activities were then compared to the activities predicted by the classification models using confusion charts. The lab-setting performance was analysed using row normalized confusion charts. With the free-living confusion charts, the cells were normalized to the total number of samples since the distribution of samples between the observed activities were not even. The activity specific sensitivity and specificity of the classification models were also added to the confusion charts of the free-living results. To show the strengths and limitations of the classification models, activities that could possibly be difficult to discriminate between were combined in a second analysis (sitting and standing to stationary and stair ascending and descending to stair walking). A sub analysis of the free-living performance on the logistics warehouse and industrial production workers was performed to investigate the impact of different activities in the validation data. BMI and age differences between the calibration and validation groups were evaluated using a two-sample t-test with a significance level of *p* < 0.05.

## Data Availability

The datasets used and/or analysed during the current study are available from the corresponding author on reasonable request.

## Abbreviations

OPA: Occupational physical activity
RF: Random forest
SVM: Support vector machine
KNN: k-nearest neighbour
BMI: Body mass index
